# Wellness Partners: Design and Evaluation of a Web-Based Physical Activity Diary with Social Gaming Features for Adults

**DOI:** 10.2196/resprot.2132

**Published:** 2013-02-01

**Authors:** Marientina Gotsis, Hua Wang, Donna Spruijt-Metz, Maryalice Jordan-Marsh, Thomas William Valente

**Affiliations:** ^1^Creative Media & Behavioral Health CenterInteractive Media Division, School of Cinematic ArtsUniversity of Southern CaliforniaLos Angeles, CAUnited States; ^2^Department of CommunicationUniversity at Buffalo, the State University of New YorkBuffalo, NYUnited States; ^3^Department of Preventive MedicineKeck School of MedicineUniversity of Southern CaliforniaLos Angeles, CAUnited States; ^4^School of Social WorkUniversity of Southern CaliforniaLos Angeles, CAUnited States

**Keywords:** physical activity, social networking, diary, game, Web-based intervention, behavior change intervention

## Abstract

**Background:**

The United States is currently in an age of obesity and inactivity despite increasing public awareness and scientific knowledge of detrimental long-term health effects of this lifestyle. Behavior-tracking diaries offer an effective strategy for physical activity adherence and weight management. Furthermore, Web-based physical activity diaries can engage meaningful partners in people’s social networks through fun online gaming interactions and generate motivational mechanisms for effective behavioral change and positive health outcomes.

**Objective:**

Wellness Partners (WP) is a Web-based intervention in the form of a physical activity diary with social networking and game features. Two versions were designed and developed for the purpose of this study—“Diary” only and “Diary+Game”. The objectives of this study included pilot testing the research process of this intervention design, implementation, evaluation, and exploring the effectiveness of social gaming features on adult participants’ physical activity and anthropometric measures.

**Methods:**

We conducted a field experiment with randomized crossover design. Assessments occurred at baseline, first follow-up (FU, 5-8 weeks after using one version of WP), and second FU (5-8 weeks of using the other version of WP). In the control condition, participants started with the “Diary” version of WP while in the experimental condition, participants started with the “Diary+Game” version of WP. A total of 54 adults (egos) ages 44-88, and their family and friends (alters) ages 17-69 participated in the study in ego-network groups. Both egos and their alters completed online surveys about their exercise habits. In addition, egos completed anthropometric measurements of BMI, fat percentage, and fat mass by bioimpedance.

**Results:**

From October 2009 to May 2010, flyers, emails, and Web advertisements yielded 335 volunteers who were screened. Rolling recruitment resulted in enrollment of 142 qualified participants in 54 ego-network groups, which were randomly assigned to a study condition. The final analytic sample included 87 individuals from 41 groups. Data were collected from December 2009 to August 2010, and data analysis was completed in 2011. Overall, the participants were given access to the intervention for 10-13 weeks. Statistical analysis suggested an increase in self-reported exercise frequency (mean days per week) from baseline (2.57, SD 1.92) to first FU (3.21, SD 1.74) in both conditions. Stronger effects were seen in the condition where Diary+Game was played first, especially in network groups with larger age variation between the alters and egos. Overall, the decrease in egos’ BMI was statistically significant from baseline to first FU, with greater decrease for those in the Diary+Game first condition (-0.26 vs -0.16 in the Diary first condition).

**Conclusions:**

The Wellness Partners program increased physical activity among participants and resulted in health benefits among the egos. Web-based diary interventions designed with social gaming features hold potential to promote active lifestyles for middle-age adults and people in their social networks.

## Introduction

The United States is currently in an age of obesity and inactivity despite increasing public awareness and scientific knowledge of detrimental long-term health effects of this lifestyle [1]. Although increases in obesity prevalence are not continuing at the same rate as in the past 10 years, the prevalence of obesity in 2009-2010 was 35.7% among adult men and 35.8% among adult women [2], meaning one third of the American adult population is obese. Recent physical activity (PA) guidelines for Americans published by the US Department of Health and Human Services recommends that adults accrue 150 minutes of moderate-intensity aerobic PA per week, or 75 minutes of vigorous-intensity aerobic PA per week to maintain healthy body weight [3].

Self-monitoring is a long-standing and effective strategy for weight maintenance and weight loss [4-7]. Empirical evidence has supported Web-based self-monitoring diaries for weight loss [8]. In addition, specific Web features (such as past journal entries, indicators of progress, and platforms for social interactions and support) are found to be significant predictors of effective Web-based interventions for weight loss and maintenance [9]. As users of social networking sites and digital games continue to grow exponentially [10,11], studies have shown high acceptability of health behavior modification programs through interactive entertainment and social media [12-14]. Social network research has shown that behaviors spread through person-to-person contact [15,16]. Interventions designed to help friends and family initiate and sustain positive behaviors can be beneficial. Bahr and others suggest that social forces can be more effective when incentives are designed to engage social networks across their ecosystem [16]. Digital games can provide compelling incentives through computer-mediated social interactions as well as virtual reward mechanisms, such as points, badges, and gifts that symbolize achievement. The Entertainment Software Association reports the average age of game players in the United States to be 37 years old [10]. However, games targeting health-related outcomes have mostly been used with younger populations for nutrition [17], cancer [18], diabetes [20], the elderly affected by stroke [21], and other acute or chronic illnesses. Studies have shown evidence of mild to moderate energy expenditure when playing active video games or exergames [22-24]. The combination of social networking influences and an intrinsically motivating game environment hold great potential to help individuals change their exercise habits and sedentary lifestyles.

Early and middle adulthood are particularly challenging periods in life, with major life transitions in education, career, and family responsibilities that lead to changes in PA [25]. Elevated levels of stress tend to increase obesity, regardless of dietary intake [26]. Exercise is an effective strategy for managing stress [27] as well as for combating obesity [28]. Young and middle-aged adults are also frequent game players [10]. Although popular game consoles such as Konami’s Dance Dance Revolution, Nintendo Wii, Sony PlayStation Move, and Microsoft Kinect can create opportunities for PA, they can also be expensive, time-consuming, and inconvenient to use. Therefore, developing interventions that promote and sustain PA with minimal technological requirements for middle-aged adults is a high public health priority.

This study had 3 objectives. First, we aimed to develop an intervention that combines social networking and digital game features in the form of a Web-based PA diary. Second, we aimed to pilot test the research process of this intervention design, its implementation, and its evaluation. We conducted a pre-study estimate of sample size and employed a rigorous procedure of recruitment, randomization, treatment, and follow-up (FU) assessments. Third, we aimed to explore the potential effectiveness of the intervention with social gaming features on participants’ PA, body mass index (BMI), fat percentage, and fat mass. We hypothesized that participants would report an increase in PA using either version of the socially networked PA diary (one with and one without game features), with a greater increase expected for those who used the version with game features in addition to social networking. Given the brief duration of the study, changes in BMI, fat percentage, and fat mass were not expected but examined.

## Methods

### Intervention Design

Wellness Partners (WP) is a Web-based intervention in the form of a PA diary with social gaming features. It was designed by the first author in consultation with the other members of the research team and with contributions from an interdisciplinary group of students and alumni. We used iterative playtesting [29] to identify critical features for behavior tracking and social game, incorporated them into the intervention design, and tested them through paper and digital prototypes. A 10-day beta testing phase was conducted with 3 pairs (N=6) of participants ages 25-44. The intervention was named Wellness Partners because the primary participants (egos) were required to partner with their family and friends (alters) and interact on the website as ego-network groups. Two versions were developed for the purpose of this study: One version with Diary only and the other Diary+Game. The following diary and social networking features were included in both versions: (1) posting updates of physical activities or setbacks, (2) sending private messages, (3) reviewing complete history of updates posted by egos and their alters, and (4) viewing display of a tag cloud of posted physical activities by all members in the egocentric network. Additional game features were included in the Diary+Game version, including: (1) displaying points as reward for updates, (2) naming of a virtual character, (3) choosing virtual locations for the virtual character’s wellness activities, (4) collecting virtual items as reward, (5) acquiring virtual character wellness animations by spending earned points, (6) including virtual character wellness activities as part of the complete history of all public updates by network members as well as their virtual characters, and (7) exchanging virtual gifts earned through some virtual character wellness activity animations (Multimedia Appendices 1 and 2). In this interventional pilot study, participants could not see each other’s virtual characters or game environment, but were able to read postings about their study partners and their virtual characters. In the Diary+Game version, the point system was balanced to enable enough points for redemption of some activities even for people who just logged on to report setbacks, which were worth less than actual PA. The point system was also balanced to allow very active users who logged on regularly and exercised regularly to be rewarded with more points. Given the expected study duration, players could visit a total of 11 locations with their virtual character, redeem up to 66 activities and earn up to 45 collectibles that could be gifted.

To facilitate the user experience, a question mark was placed next to each key feature on the WP website with descriptions in a pop-up window. Contact points for technical support were provided on the WP study website frequently asked questions page with an email and a phone number, a hyperlink to the bug report was included in the daily email reminders, and an open-ended question was offered for comments and suggestions at the end of each online survey. The qualitative feedback was obtained through participant interviews.

### Research Design

**Figure 1:**
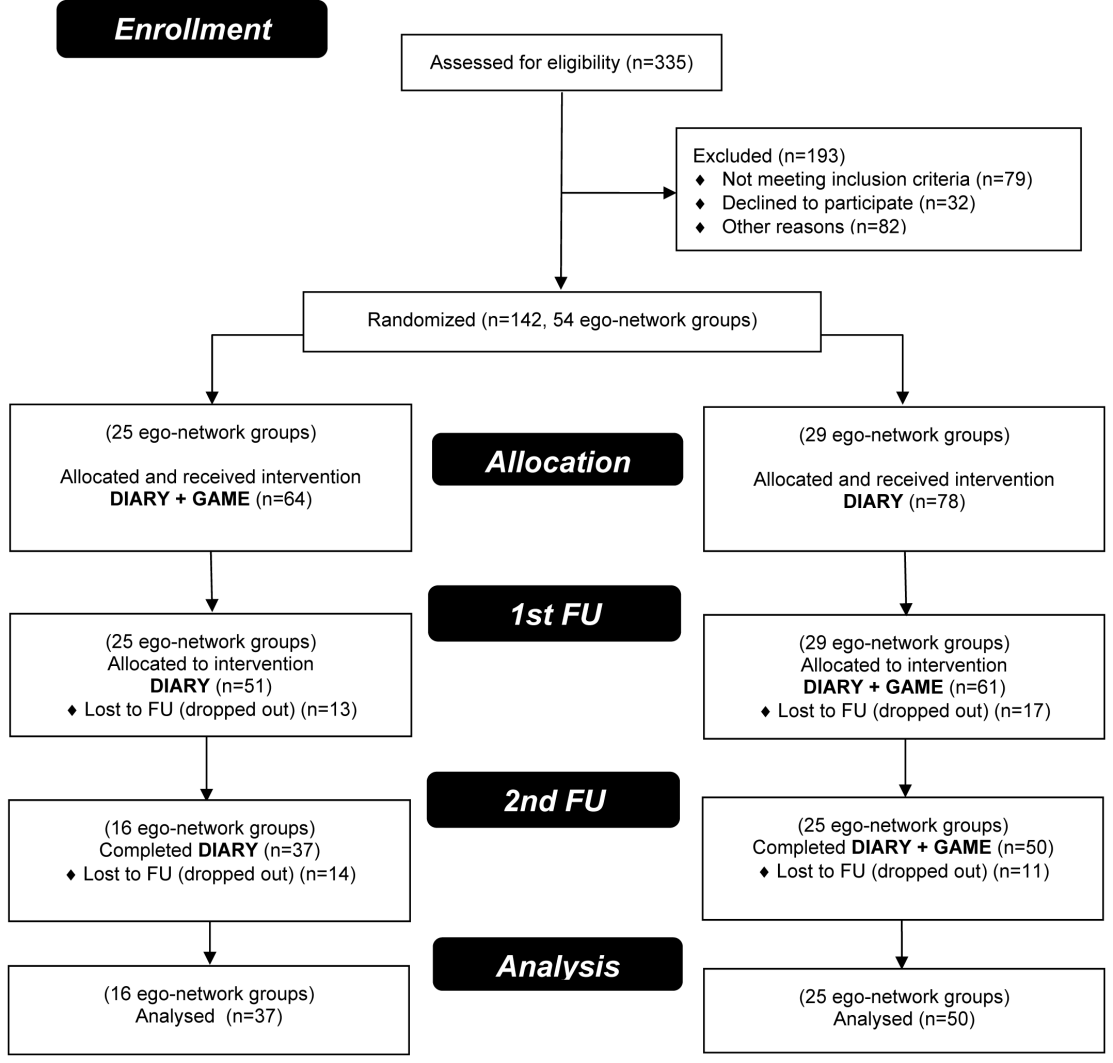
CONSORT 2010 flow diagram. Of these 54 ego-networks, 12 groups (28 people) failed to complete all survey measurements and were dropped from the analyses (n=142, 22.3% attrition). In addition, 27 participants dropped out from the study, leaving a final analytic sample of 87 individuals from 41 groups (n=142, 38.7% attrition).

This study used a randomized crossover design with two arms. Assessments occurred at baseline, at first FU (5-8 weeks) and at second FU (10-13 weeks) (Figure 1). From October 2009 to May 2010, rolling recruitment was used to screen and enroll qualified volunteers in ego-network groups. The study was advertised via flyers, emails and on a website. Each ego-network group was randomly assigned to start with one of the two versions of the intervention (Diary or Diary+Game). Data were collected from December 2009 to August 2010. Data analysis was completed in 2011.

### Participants and Procedure

In coordination with the Center for Work and Family Life at USC, a mass email announcement and 12,000 paper advertisements were sent to all university staff directing volunteers to an email, voicemail, and website. Before they were contacted for screening, volunteers had to fill out an online consent for contact form. Volunteers were screened over the phone for the following inclusion/exclusion criteria: (1) age between 25 and 44, (2) English fluency (rating of at least 7 on a 10-point scale), (3) employment status (required to be university staff, employed full-time, or part-time), (4) daily Internet access, (5) cell phone ownership, (6) no current participation in any other formal studies of weight loss or healthy lifestyle, (7) no prior participation in WP, and (8) report of having a social support network containing at least 4 people.

Qualified volunteers became egos in the study and were instructed to fill out the online referral form, nominating members of their social network to join their ego-network group and enroll in the study as their alters. Volunteers who were referred by egos were asked to fill out the online consent for contact Form. Potential alters were screened over the phone for the following inclusion/exclusion criteria: (1) age between 12 and 85, (2) English fluency (rating of at least 7 on a 10-point scale), (3) daily Internet access, and (4) no prior participation in WP.

Employment by USC was not a criterion used for alters because we wanted to encourage participants to enroll any member of their friends or family, regardless of employment and where they lived. Egos had to be willing to come to campus for anthropometric measurement, but since alters did not have to be seen in person by our staff they could be located anywhere.

When an ego and at least one alter mutually confirmed their participation in the study, they were each asked to review the online informed consent form and complete a baseline survey. Parental consent was obtained for minors. Then, the ego was scheduled for an in-person baseline anthropometric assessment before the entire ego-network group was assigned a group ID and randomized (as a group) in blocks of 10 to begin one of the two study conditions. A random number generator was set up with a minimum value of 1 and a maximum value of 10 where odd numbers were assigned to Diary+Game and even numbers were assigned to Diary until one of the two conditions reached 5 ego-network groups and the rest were assigned to the other condition. These steps were repeated as the group sample exceeded 10, 20, 30, etc.

Upon completion of baseline assessment milestones, participants were emailed a user name and password with user instructions and encouraged to log on to the WP website everyday (Multimedia Appendix 3). Additional alters could be added to the ego-network group upon successful completion of phone screening, online informed consent, and baseline survey. After each ego completed first FU anthropometry and online survey, the entire ego-network group was switched to the other condition with email notification. First FU occurred between weeks 5 and 8 of the study and second FU occurred approximately between weeks 10 and 13.

**Figure 2:**
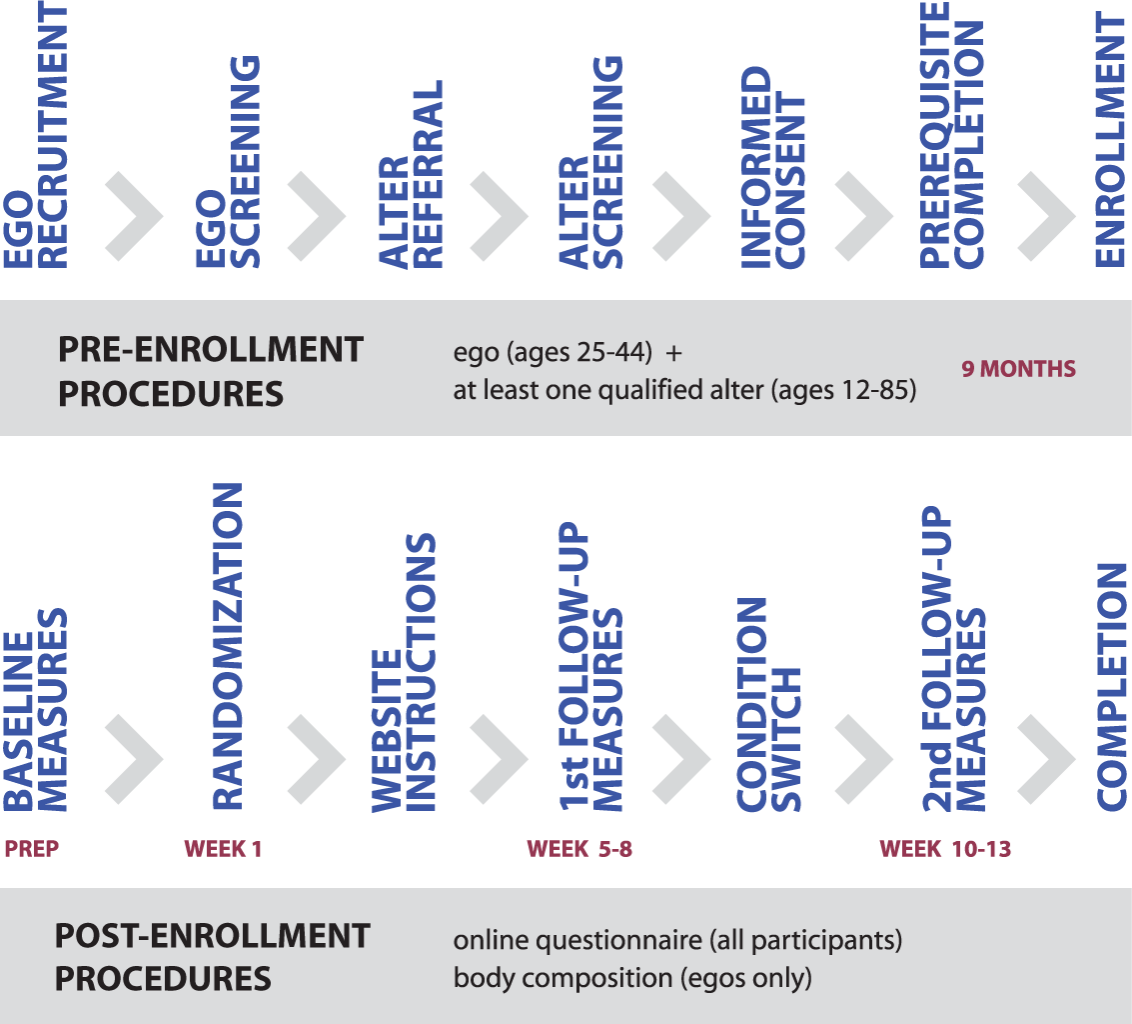
Pre-enrollment and post-enrollment study procedures.

Incentives were provided after completion of each data collection milestone—$10 for assessment at baseline, $10 for first FU, and $25 for second FU. All egos and up to their first 5 alters were mailed checks and minors were mailed gift certificates. This study procedure (Figure 2) was approved by the Institutional Review Board at USC.

### Main Outcome Measures

The main outcome variables for this study included self-reported exercise frequency of all participants and anthropometric measures of all egos. PA was measured using one item that asked, “on average, how often do you exercise (minutes per day, days per week)” as used in previous studies [30]. Egos’ anthropometric data included BMI, fat percentage, and fat mass, collected by trained staff at the Center for Work and Family Life or at the participants’ office, using a body composition analyzer (Tanita TBF-215GS, TBF-300A, Japan) and a standardized protocol.

### Statistical Analysis

Means were reported first by survey wave and condition (Diary vs Diary+Game) to establish data trends. *T* tests comparing baseline and FU scores were conducted to determine if the interventions changed PA and body composition. Random effects lagged regression models were used to determine if changes were influenced by individual participants or study characteristics. A variable indicating the order of study condition (Diary or Diary+Game first) was included in the analyses to determine if the effects were sensitive to the specific starting condition of the ego-network groups. All analyses were completed using STATA version 11.1 (StataCorp LP, Texas, USA) with alpha set at *P*≤.05 for significance tests on PA and *P*≤.10 for significance tests on egos’ BMI, fat percentage, and fat mass.

### Power Analysis

Using a general power equation with standard assumptions and reaching a medium effect size [31,32], we calculated that we would need 30 groups per condition to detect significant changes in PA with the *P* value set at .05. As shown in Figure 1, our sample size and attrition rate did not meet the expected estimates. Therefore, these results should be interpreted with caution.

## Results

### Participant Demographics

The initial sample included 54 egos and 88 alters that added up to a total of 142 individual participants in 54 ego-network groups at baseline (Figure 1). Egos recruited an average of 1.63 alters to be their “wellness partners”. The minimum and modal group size was 2 (63%) with 13 groups including 3 people (24.1%) and maximum group size was 8. Over the course of the study, 49 people dropped out, and 6 people were excluded due to technical errors, leaving an analytical sample of 87 individual participants in 41 ego-network groups.

Table 1 reports the sociodemographic characteristics and descriptive statistics for the entire sample, participants lost to FU, participants lost to technical error, the analytic sample, and its breakdown by condition. In the entire sample, there were more female participants (95/142, 67.6%), more college graduates (103/142, 72.5%), and ethnic diversity (approximately 25/142, 18% were Asian or Asian American, 40/142, 28% were Hispanic/Latino) with an average age of 35.6 years. Participants who dropped out at FU were significantly more likely to be alters and have higher estimated BMI at baseline than those who completed the entire study. Comparisons on the analytic sample between conditions (Diary vs Diary+Game first) suggested that participants in both conditions were similar except that those in the Diary+Game first condition were younger (32.0 vs 38.7 years) and had a younger group average age (32.4 vs 38.9 years) than those in the Diary first condition. There were no statistically significant differences between egos and alters on these characteristics though alters were slightly more likely to be male, White, older, and less educated (results not shown).

**Table 1:** Sociodemographic characteristics of study participants by condition.

		Total	Lost to FU	Analytic sample	Diary first	Diary+Game first
Number of participants		142	55	87	49	38
Male, n (%)		46 (32.4)	17 (30.9)	29 (33.3)	14 (28.6)	15 (39.5)
College graduate, n (%)		103 (72.5)	35 (63.2)	73 (83.9)	39 (79.6)	34 (89.5)
Asian ethnicity, n (%)		25 (17.6)	5 (9.1)	20 (23.0)	9 (18.4)	11 (29.0)
Hispanic ethnicity, n (%)		40 (28.2)	20.2 (36.4)	20 (23.0)	10 (20.4)	10 (26.3)
Mean age (SD) years		35.56 (9.51)	35.24 (10.05)	35.80 (9.21)	38.69 (9.21)	32.00 (7.82)
Alters, n (%)		89 (62.7)	42 (76.4)	47 (54.0)	25 (51.0)	22 (57.9)
**Ego-network characteristics**						
	Group size	3.11	3.07	3.14	3.14	3.13
	Mean group age in years (SD)	35.56 (5.72)	34.8 (6.04)	36.1 (5.5)	38.9 (6.6)	32.4 (4.2)
**Outcomes**						
	Mean days of PA per week (SD)	2.49 (1.87)	2.35 (1.86)	2.57 (1.89)	2.59 (1.83)	2.55 (1.98)
	Estimated baseline BMI	26.97 (6.00)	29.06 (7.82)	26.30 (5.17)	26.52 (4.97)	26.02 (5.47)

### User Logins

The WP server kept track of user activities. Overall, participants were given access to the intervention for 10-13 weeks. Our server data suggested that among participants in the analytical sample, in general, they accessed the WP website every other day, with the number of total logins ranging from 1-102 (mean 38.00, SD 22.31), the number of days they logged in ranged from 1-81 days (mean 32.75, SD 18.32), and the most popular times of the day they logged in were 9 am to 12 noon (mean 8.68 times), 6 pm to 9 pm (mean 7.32 times), and 9 pm to 12 midnight (mean 7.11 times).

### Intervention Outcomes

Over time, mean number of self-reported days of exercise per week at baseline, first FU, and second FU were: 2.57, 3.21, and 3.23, respectively.

**Figure 3:**
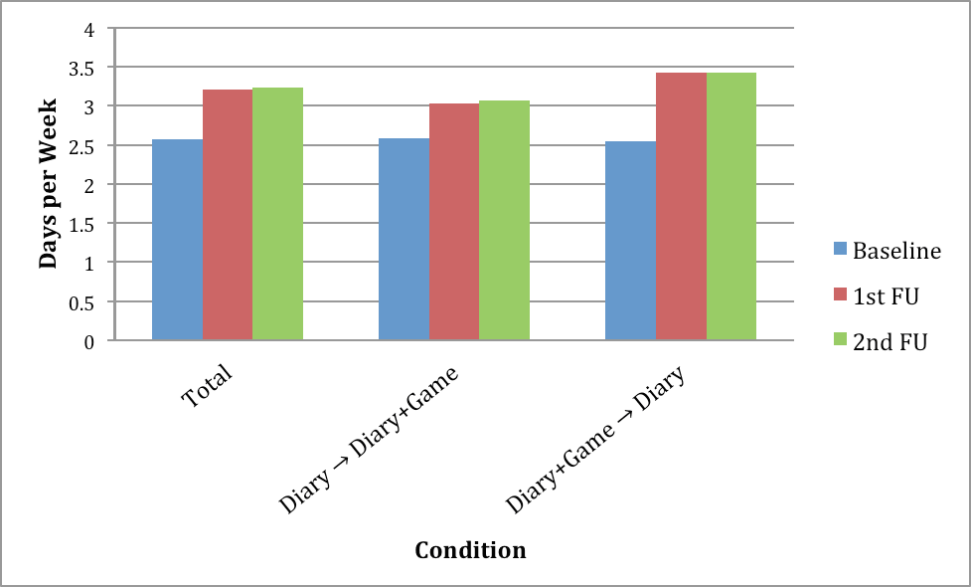
Exercise days per week at baseline, first, and second FU overall (total) and by Diary first versus Diary+Game first. The exercise frequency increase was greater in the Diary+Game first condition.

**Figure 4:**
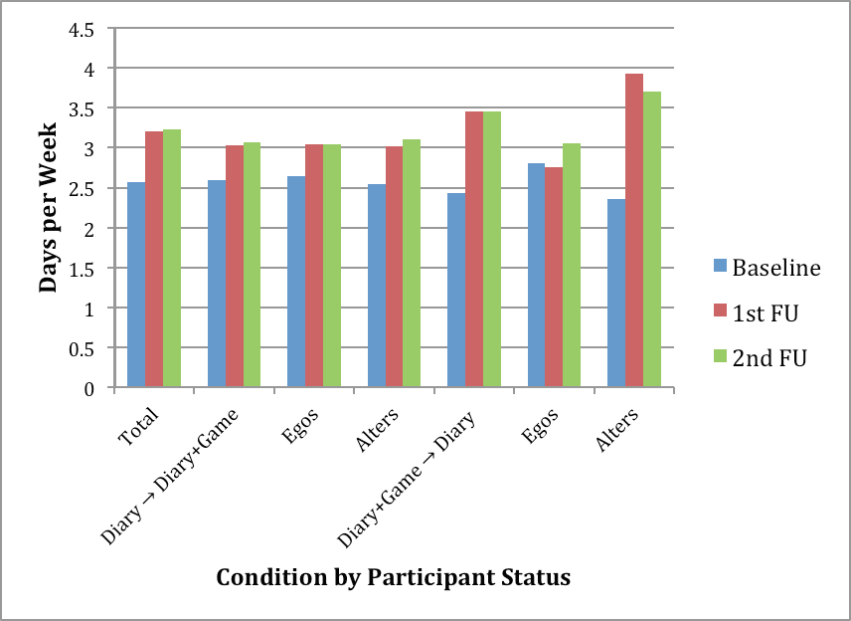
Exercise days per week at baseline, first, and second FU overall (total), by study condition (Diary first vs Diary+Game first), and by participant status (ego vs alter). The exercise frequency increase was greatest for alters in the Diary+Game first condition (1.57 days per week).

These overall trends, however, mask differences by condition (Diary vs Diary+Game first), and by participant status (ego vs alter). Figure 3 and Table 2 show self-reported exercise frequency at baseline, first, and second FU by study condition. In both conditions, there was an increase from baseline to first FU, yet the increase was lower in the Diary first condition (0.44 days vs 0.88 days per week). The Diary+Game first condition reported lower baseline exercise frequency (2.55 days per week) and higher exercise frequency at FU (3.43 days per week). Participant status may have influenced increase in PA, as shown in Table 2 and Figure 4. Increases were similar for egos and alters in the Diary first condition, and there was a trend for greater increase in the alters in the Diary+Game first condition. These differences did not attain statistical significance in the analysis of variance (*F*_2_=2.56, *P*=.08).

**Table 2:** Self-reported days per week exercised by study condition and participant status.

		Baseline Mean (SD)	1^st^ FU Mean (SD)	2^nd^ FU Mean (SD)
Total		2.57 (1.92)	3.21 (1.74)	3.23 (1.68)
**Diary → Diary+Game**				
	Total	2.59 (1.83)	3.03 (1.64)	3.07 (1.55)
	Egos	2.64 (1.96)	3.04 (1.52)	3.04 (1.57)
	Alters	2.54 (1.73)	3.02 (1.78)	3.10 (1.55)
**Diary+Game → Diary**				
	Total	2.55 (1.98)	3.43 (1.87)	3.43 (1.89)
	Egos	2.81 (2.14)	2.75 (1.84)	3.06 (2.17)
	Alters	2.36 (1.89)	3.93 (1.76)	3.70 (1.65)

A random effects regression model was calculated using the ego-network group (the ego and their alters) as the random effects and sociodemographic variables (sex, education, ethnicity, age, estimated BMI, mean group age, and SD group age) were entered as controls, which may also mask intervention effects. The results in Table 3 represent unstandardized random effects coefficients indicating the amount of change in self-reported PA for each unit change in the corresponding independent variable. For example, a one unit change in self-reported PA at baseline is associated with a 0.61 increase in self-reported PA at first FU. The first set of coefficients include only main effects, the second column (for each follow up period) report the main effects and include the interaction term of being an alter and being in the Diary+Game condition first.

As expected, baseline exercise frequency was strongly and significantly associated with exercise frequency at both first and second FU. The only significant predictor of increased self-reported PA at first FU was group age variation, indicating that groups composed of people of varying ages were more likely to increase their PA than those with less variation.

**Table 3:** Random effects regression coefficients on number of days per week exercised at FU.

		Number of days exercised 1^st^ FU Regression coefficient (*P* value)		Number of days exercised 2^nd^ FU Regression coefficient (*P* value)	
		Main effects only	Interaction included	Main effects only	Interaction included
1^st^ FU		NA	NA	0.52 (<.001)	0.49 (<.001)
Baseline		0.61 (<.001)	0.64 (<.001)	0.35 (<.001)	0.38 (<.001)
Diary+Game first		0.55 (.08)	-0.30 (.40)	0.23 (<.35)	0.01 (.89)
Male		0.45 (.11)	0.37 (.14)	-0.19 (.45)	-0.19 (.16)
Education		0.59 (.09)	0.52 (.07)	0.24 (.25)	0.24 (.61)
Asian ethnicity		-0.40 (.12)	-0.35 (.15)	0.20 (.27)	0.20 (.36)
Hispanic ethnicity		0.42 (.31)	0.37 (.37)	0.79 (.004)	0.78 (.004)
Age		0.03 (.34)	0.05 (.09)	0.04 (.017)	0.04 (.011)
Alter (vs ego)		0.68 (.035)	-0.15 (.65)	0.18 (.47)	-0.03 (.93)
**Ego-network characteristics**					
	Group size	-0.03 (.76)	-0.03 (.72)	-0.04 (.25)	-0.05 (.25)
	Mean group age	-0.01 (.70)	-0.02 (.57)	-0.02 (.33)	-0.03 (.30)
	Group age SD	0.07 (.012)	0.06 (.023)	0.01 (.53)	0.01 (.54)
**Outcome**					
	Estimated baseline BMI	-0.04 (.26)	-0.04 (.18)	0.01 (.79)	0.0 (.88)
**Condition by status**					
	Diary+Game alter interaction	not applicable	1.74 (.009)	not applicable	0.48 (.26)
	Adjusted R^2^	57%	63%	79%	79%

To test the combined effects of study condition and participant status, an interaction term of condition (Diary+Game first) and being an alter (rather than an ego) was constructed. Model estimates showed that the covariates remained mostly unchanged and the interaction term was statistically significant indicating that alters in the networks that started with the Diary+Game condition increased their self-reported PA significantly compared to egos and those in the Diary first condition.

Whether the changes in self-reported behavior translated into changes in anthropometric measures (ie, BMI, fat percentage, fat mass) for the egos was also examined. Anthropometric measures were only available for egos, and of the 40 egos, 34 had measurements at all 3 waves paired *t* tests on 3 measures of adiposity were conducted jointly and separately by condition, and given the smaller sample size, *P* values less than .10 were considered significant. Table 4 shows that, overall, there was a significant decrease in BMI from baseline to first FU, which was driven primarily by a decrease in BMI for the Diary+Game first condition. Overall, neither fat percentage nor fat mass decreased statistically, although there was a trend towards significant decreases in both fat percentage and fat mass in the Diary+Game first condition. These anthropometric measures showed a decrease in BMI and body fat for egos from baseline to first FU for those who received the Diary+Game first. Anthropometric measures at the second FU did not change for the egos.

**Table 4:** Anthropometric measures by study condition.^a^

		Baseline	1^st^ FU	Difference	*P* value	Baseline	2^nd^ FU	Difference from 1^st^ FU	*P* value
N		38	38			33	33		
**BMI**									
	Total	28.02	27.83	-0.19	.02	28.09	27.99	-0.10	.23
	Diary → Diary+Game	28.02	27.86	-0.16	.10	28.05	27.94	-0.10	.28
	Diary+Game → Diary	28.03	27.77	-0.26	.07	28.16	28.05	-0.10	.32
**Fat percentage (pounds)**									
	Total	30.95	30.84	-0.12	.40	30.63	30.51	-0.12	.39
	Diary → Diary+Game	30.70	30.94	0.25	.64	30.27	30.54	0.27	.66
	Diary+Game → Diary	31.34	30.67	-0.67	.11	31.13	30.49	-0.64	.12
**Fat Mass (pounds)**									
	Total	54.00	53.51	-0.49	.27	53.58	52.97	-0.61	.25
	Diary → Diary+Game	54.17	54.21	0.05	.52	53.53	53.48	-0.05	.48
	Diary+Game → Diary	53.74	52.43	-1.31	.09	53.66	52.29	-1.37	.16

^a^ N=38, Diary → Diary+Game, n=23; Diary+Game → Diary, n=15; N=33, Diary → Diary+Game, n=19; Diary+Game → Diary, n=14.

We also compared egos’ and alters’ self-reported health status by examining responses to one question at baseline, “in general, would you say your health is…” (1 = excellent and 5 = poor). Alters tended to report significantly better health status than egos (2.48 vs 2.81; *F*_1,140_=3.84; *P*=.052).

## Discussion

### Major Findings

To our knowledge, this is the first study to design, develop, and evaluate a Web-based PA diary intervention for adults that focused on the effects of social gaming. The concept of using game mechanics for motivating real world behaviors is now known as “gamification” [33]. We used a rigorous procedure for rolling recruitment and enrollment and collected data from different sources. Our findings demonstrated the potential of combining influence from close social ties and social gaming practices to promote an active and healthy lifestyle. During the course of a year, 87 middle-aged adults along with their family and friends formed 41 ego-network groups and participated in the WP program. The results showed modest, but statistically significant increases in self-reported PA, especially among alters, ego-network groups of larger age variation, and participants in Diary+Game first condition. Overall, the decrease in egos’ BMI was statistically significant from baseline to first FU, with larger effects for those in the Diary+Game first condition. Interestingly, this effect did not weaken or improve at second FU, suggesting that the second condition served to maintain weight loss regardless of condition. The decreases in egos’ fat percentage and fat mass were not statistically significant overall from baseline to first FU, although there was a trend for significance for those in the Diary+Game first condition. Overall, the intervention effects were stronger in the first period, with the second period serving as a maintenance phase.

The greater effectiveness of the social gaming features among alters is an intriguing finding. Alters were not different from each other on demographic characteristics. Their increased PA may have been prompted by a desire to have their egos look good in the study. Alternatively, they may have genuinely been more motivated to increase their PA because their family or friends recruited them. A third possibility is that the egos selected the alters purposely as someone who either would, or needed to, change their own behavior. However, the finding that alters reported significantly better perceived overall health than egos suggests that although individuals recruited others who were similar on sociodemographic characteristics, they may have reached out to “healthier” friends and family who could “help” them rather than try to positively influence others themselves. This pattern may have implications for recruitment of PA studies that have a social networking component.

The effectiveness did not vary by sociodemographic characteristics, indicating that the intervention was just as effective among younger and older participants, those of different ethnicities, with different educational levels, and different genders. This indicates that the social gaming features had broad appeal among this selected but varied population. This is encouraging considering the rudimentary nature of the game mechanics and interface. Interactive entertainment applications can be costly to develop and this study demonstrated that even basic features could provide behavior modification potential for increasing PA.

### Limitations

#### Technical Problems

Any new technology-based innovative interventions will have to face technical challenges. Engagement and retention of participants in this study may have been influenced by software problems and limitations they encountered. A number of projected features including a Twitter-enabled reporting function were not deemed stable enough for the main study after beta testing. Some software problems were known from beta testing, such as slow loading. Some participant activities did not get logged on the WP website due to a persistent random software bug that could not be corrected while the study was in progress. During one particular week, no email reminders were sent out to the participants. Upon quality assurance review of data, we discovered that the conditions of a few ego-network groups were not switched after first FU, and were excluded from the analytical sample (Figure 1).

#### Game Design

An additional limitation of the study related to accumulation of points earned from exercise in the Game+Diary version of the software. Participants could report an infinite number of PA or setbacks per day, but they were awarded points only for the first report within a 24-hour period. Several participants voiced frustration about this as a usability problem within the first few weeks of the study. We had not anticipated that participants would login more than once per day to report. We revised the reward schedule to 12 hours while the study was ongoing. This means that some of the participants earlier in the study received fewer points and may have not enjoyed the experienced as much as later participants. No other revisions were made to the point system as seen in (Table 5), which was not disclosed to participants.

**Table 5:** Wellness Partners Game+Diary point reward system.

		Number of participants	>15 mins	<15 mins	<30 mins	<45 mins	<60 mins	<75 mins	<90 mins
**Points awarded for individual (solo) activities**									
	Light	1	1	3	5	7	8	9	9
	Medium	1	1	8	10	12	15	17	17
	Heavy	1	1	12	15	18	21	24	24
**Points awarded for group activities (given to all reported activity participants)**									
	Light	2	1	1	3	2	2	2	2
		3	2	2	4	3	3	3	3
		≥4	3	3	5	4	4	4	4
	Medium	2	1	1	2	2	2	1	1
		3	2	2	3	3	3	1	1
		≥4	3	3	4	4	4	1	1
	Heavy	2	1	1	3	3	2	1	1
		3	2	2	4	4	3	2	2
		≥4	3	3	5	5	4	3	3
**Points awarded for player activity posting**									
	First activity ever					5			
	Second activity of the day					2			
	First setback of the day					1 (0 points thereafter)			

We did collect feasibility data in this study, which includes FU survey questions on usability and evaluation of various features in each version of the intervention, user technology literacy (eg, prior gaming experience and participation on social networking websites), daily software bug reports, and 20 semi-structured, in-depth interviews [34]. However, here we focus on reporting the intervention and research design as well as major findings in the evaluation in this article and provide detailed information and analysis of feasibility data in a separate paper with insights for future development of such applications.

#### Outcome Measurement

Another limitation of this study is the use of a single self-report item measure of PA, which did not include accelerometry. Given limited resources at the time of study design, we determined that self-reported PA combined with objective measures of outcome, such as BMI, fat percentage, and fat mass would be adequate for this interventional pilot study.

### Directions for Future Research

A future version of this study would include a number of revisions in both versions of the intervention and study procedures. The first proposed revision relates to mobile access. Due to the exploratory nature of the intervention and the wide variety of socioeconomic characteristics of the intended audience, it was determined that the lowest common denominator technology would be a computer with a Web browser that participants could access at least once per day at home or at work. The Diary version of the intervention was compatible with mobile phones that had full Web browser support and the Diary+Game version was only compatible with Flash-enabled mobile phones. Given current smartphone ownership and social networking website diffusion, a revision of the software would be developed as a mobile app as well as a website to provide participants easy access.

The second revision would be the study design. This study used a crossover design without a washout period. Ego-network groups switched conditions within 24 hours of egos completing their first anthropometry FU. Although a washout period is common for pharmaceutical studies, this study did not have a washout period for several reasons. One secondary objective for this study was to determine whether a simple game could help jumpstart PA self-reporting and/or make it less tedious. Observing the immediate effects of ending the game was therefore important. In future analyses, researchers might also compare participants’ satisfaction with the website versions and see whether greater affinity for either version (with or without game features) resulted in changes in usage behavior and/or changes in PA or other measures. For example, participants may report dislike of a version, yet they may benefit from adherence to it and not realize it is working for them until the program is swapped for something else. In addition, this study focused on the evaluation of the effectiveness of social gaming features as opposed to social networking only. Future studies may consider adding a control group with no intervention, or a wait list control group.

The third revision relates to the research procedure. A fully powered study based on the criteria described under the section Power Analysis would have to address both adequate enrollment and strategies for minimizing attrition. Given budgetary constraints, a manual rolling recruitment and data collection process was designed and adopted in this study. It was complicated to manage and required too many steps, which was a burden for study coordinators and potential participants. For example, egos whose alters delayed contacting us may have experienced higher frustration, which contributed to dropout rates. Future attempts of a similar approach could benefit from an automated content management system for participant recruitment, screening, and frequent reminders to egos about inviting unlimited alters.

The fourth proposed revision is on the objective measurement. Scheduling in-person anthropometric measurements was another challenge to participant retention. Since switching of the condition followed the egos’ schedule of data collection, if an ego delayed completion of anthropometry or the online survey, the group would stay longer in their initially assigned condition. Future studies should take advantage of the more widely available free accelerometer mobile apps and obtain anthropometric measures from both egos and alters rather than relying on self-reports.

Finally, the fifth revision is regarding sampling. Implementing the study in a community setting, rather than a workplace setting, could be more convenient for some participants. Conducting the study with a population sample, rather than an organizational one, would provide even greater generalizability.

### Conclusion

WP was designed as a Web-based PA promotion diary with social gaming features for adults. The intervention design requires an iterative process to ensure the quality of basic features. Rigorous research design and study procedure are needed to implement and evaluation the intervention. The game features included in the Diary+Game version of WP did not have a great variety or superb sophistication, greater depth and length of play would be required to produce stronger effects and sustain long-term changes. However, our empirical findings suggest that the Diary+Game version had stronger effects on BMI and body fat, with a trend for increased PA. Results were modest, yet surprising, given the short study duration and the absence of a nutritional intervention. This suggests that interventions with social gaming features hold promise in the battle against obesity.

## Multimedia Appendix 1

Video documentation of Wellness Partners website (Game Version).

## Multimedia Appendix 2

Art asset documentation of Wellness Partners (Game Version).

## Multimedia Appendix 3

Documentation of instructions emailed to Wellness Partners study participants with their username and password. Version of instructions and version of website participants received access to was determined by random assignment of ego-network groups.

## Abbreviations

BMI: body mass index
FU: follow-up
PA: physical activity
USC: University of Southern California
WP: Wellness Partners
