# Supplementary material for: Wellness Partners: Design and Evaluation of a Web-Based Physical Activity Diary with Social Gaming Features for Adults
Source: JMIR Res Protoc. 2013 Feb 1;2(1):e10. doi: 10.2196/resprot.2132 (PMC3628151; doi:10.2196/resprot.2132)
Supplement: Supplementary file 2 [file resprot_v2i1e10_app2.pdf]

Multimedia Appendix 3

Wellness Partners (Game Version)

Character LOCATIONS

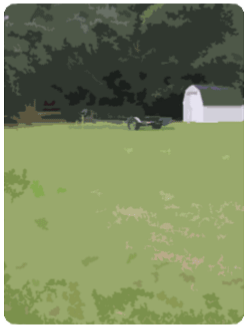

My Backyard

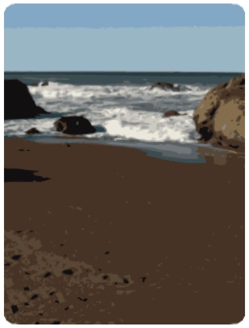

Moonstone Beach

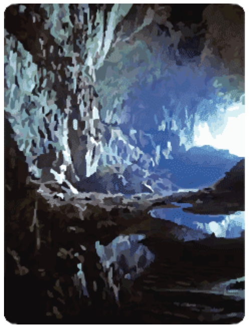

Crystal Cave

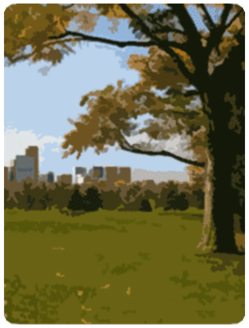

Wellnesstown City Park

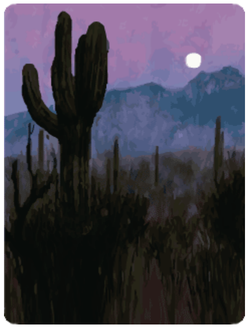

Blue Coyote Desert

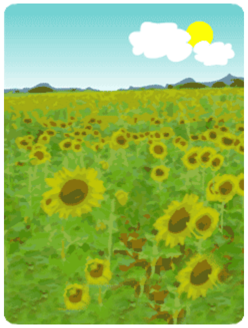

Sunflower Valley

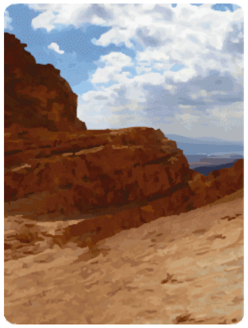

Red Cactus Mountain

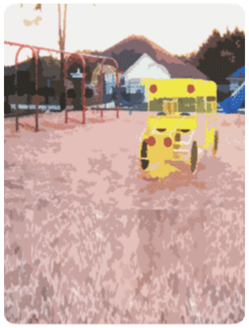

Sunny Hill Playground

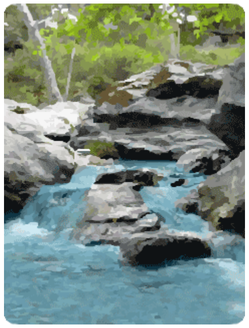

Shimmering Rock River

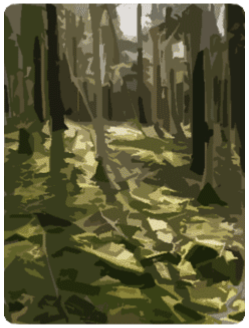

Golden Pines Woods

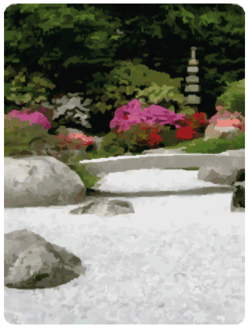

Zen Garden

MEMORIES that resulted from ACTIVITIES (points spent to redeem)

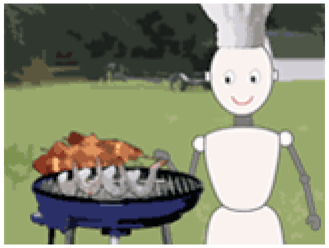
Have a BBQ (15)

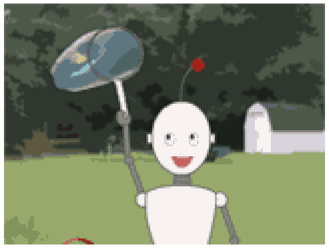
Play with Bubble Wand (20)

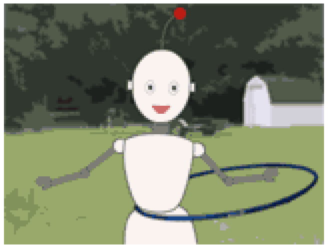
Hulahoop (10)

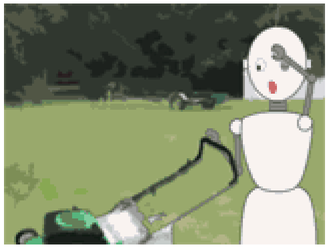
Mow the Lawn (5)

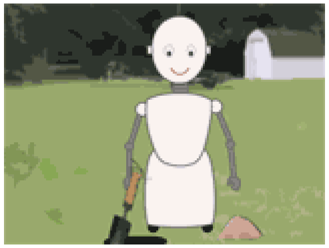
Plant a Seed (10)

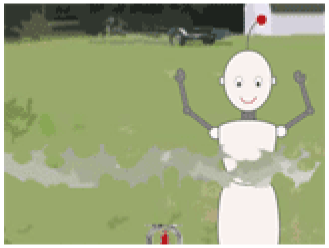
Play in the Sprinkler(25)

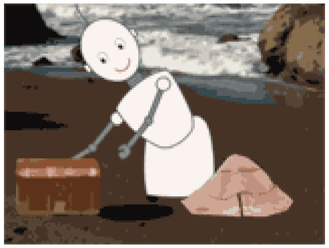
Bury Treasure (25)

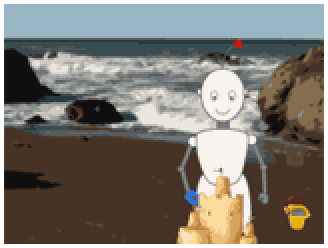
Make a Sand Castle (10)

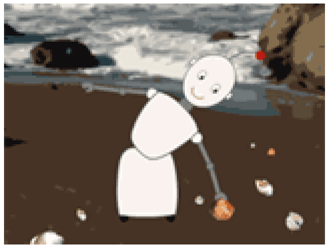
Collect Seashells (5)

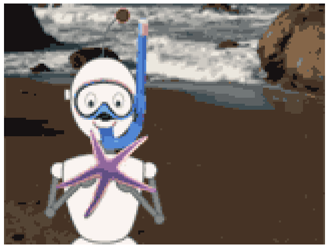
Go Snorkeling (20)

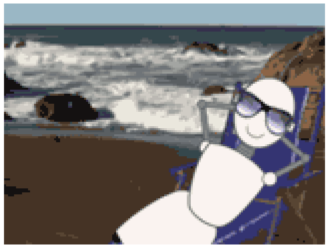
Sunbathe (10)

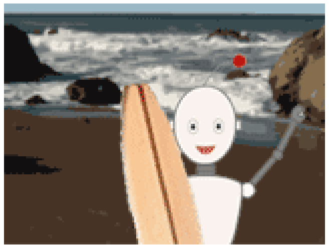
Go Surfing (15)

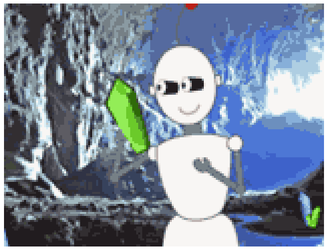
Examine the Crystals (15)

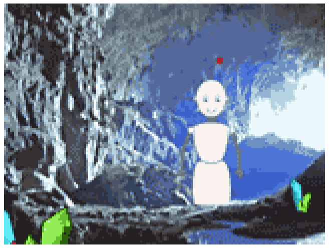
Explore (10)

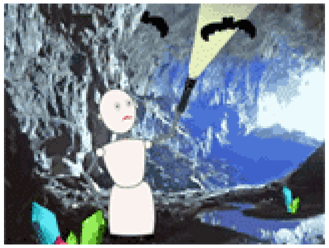
Shine a Flashlight (5)

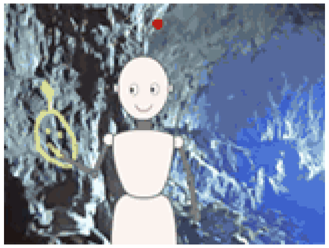
Make a Cave Painting (25)

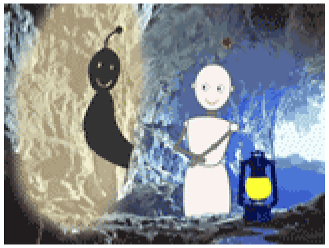
Make Shadow Puppets (10)

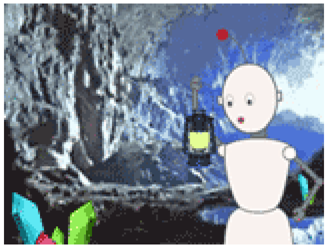
Use a Lantern (10)

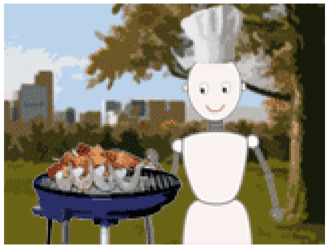
Have a BBQ (15)

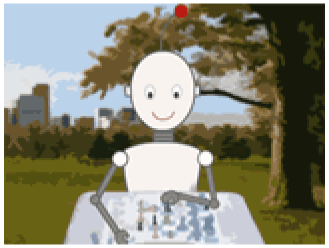
Play Chess (20)

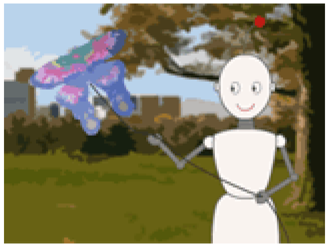
Fly a Kite (10)

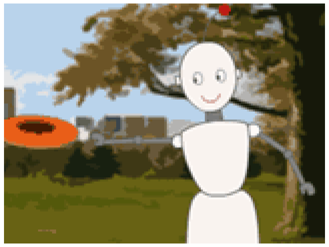
Toss a Frisbee (5)

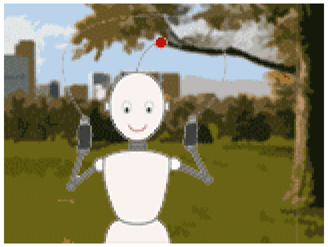
Jump Rope (10)

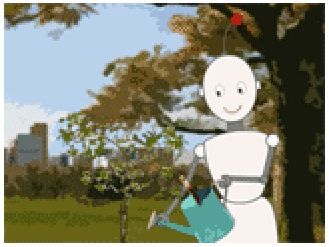
Plant a Tree (25)

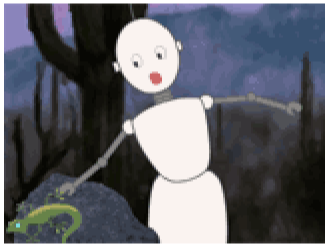
Catch a Lizard (20)

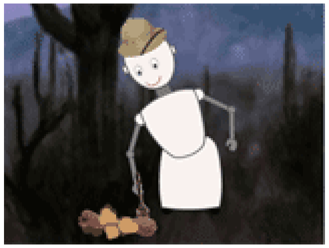
Find a Fossil (15)

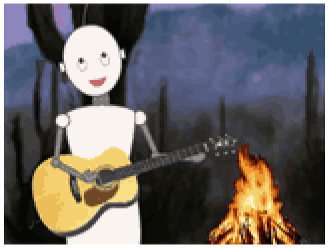
Play Guitar (25)

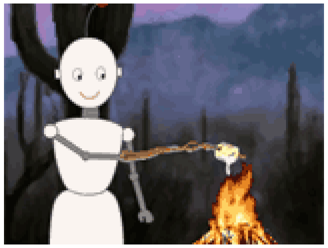
Roast Marshmallows (5)

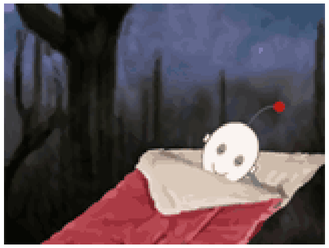
Sleep under the Stars (10)

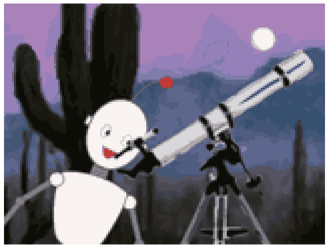
Stargaze (10)

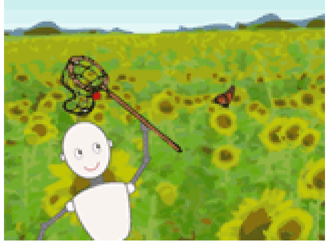
Catch Butterflies (25)

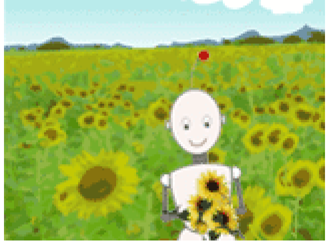
Make a Flower Necklace (15)

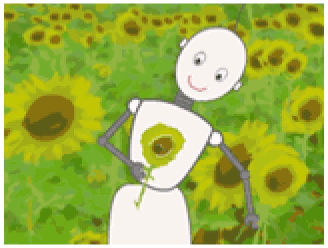
Pick Flowers (10)

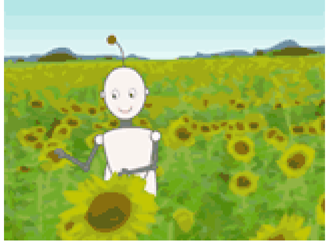
Pick Sunflower Seeds (5)

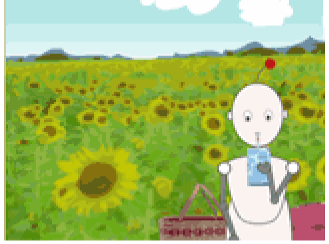
Go on a Picnic (20)

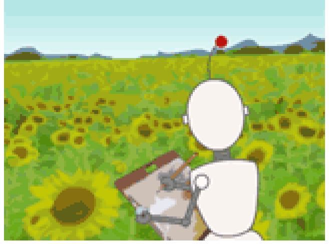
Sketch a Picture (10)

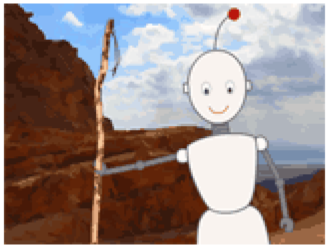
Go on a Mountain Hike (5)

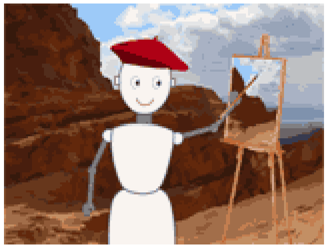
Make a Landscape Painting (10)

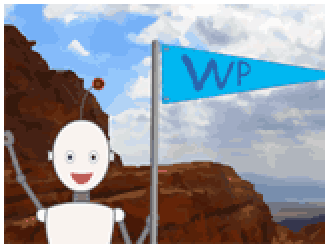

Plant a Flag (25)

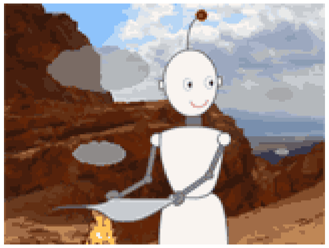
Make Smoke Signals (15)

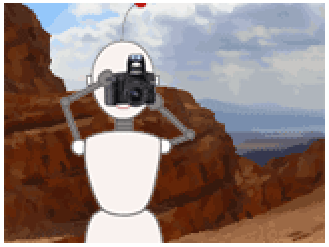
Take a Picture (10)

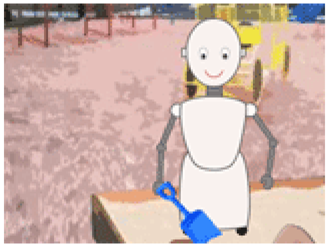
Dig in the Sandbox (20)

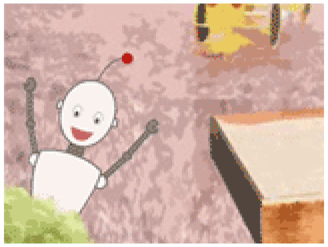
Play Hide and Seek (5)

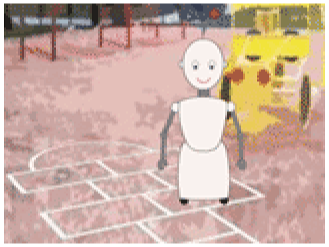
Play Hopskotch (15)

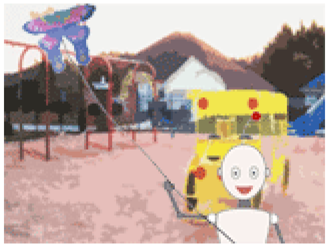
Fly a Kite (10)

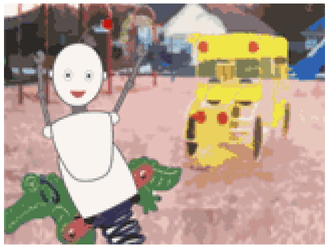
Ride a Spring Animal (25)

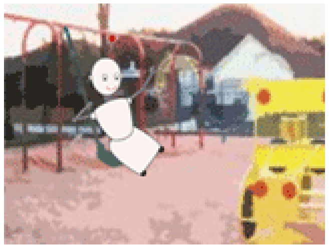
Swing (10)

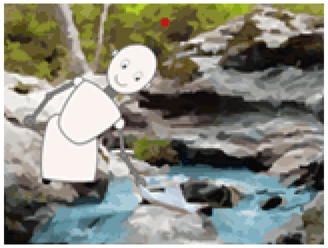
Make a Paper Boat (20)

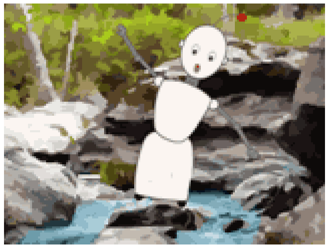
Cross the River (10)

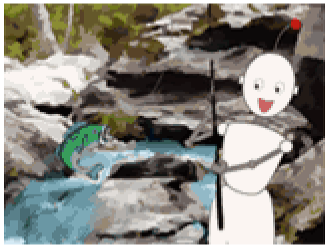
Go Fishing (15)

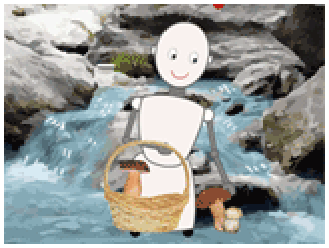
Collect Mushrooms (5)

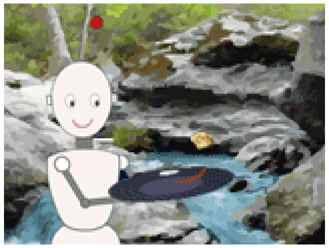
Pan for Gold (10)

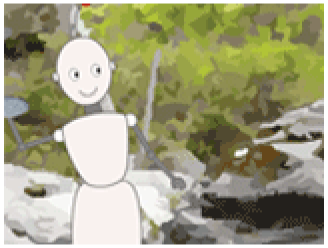
Skip a Stone (25)

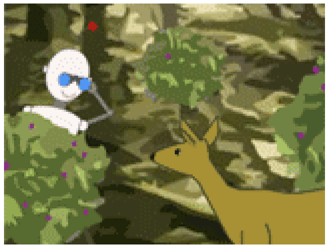
Track an Animal (25)

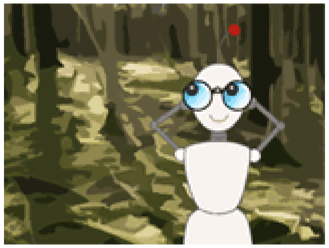
Go Bird Watching (15)

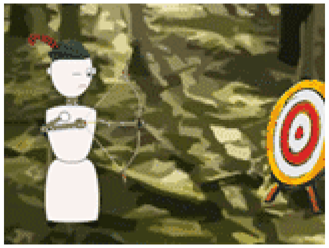
Fire a Bow and Arrow (20)

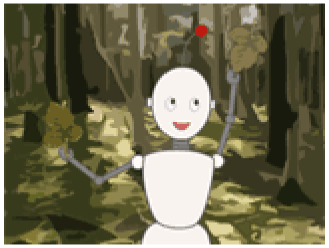
Catch Leaves (10)

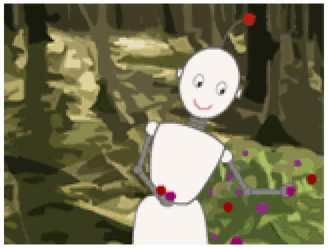
Pick Berries (5)

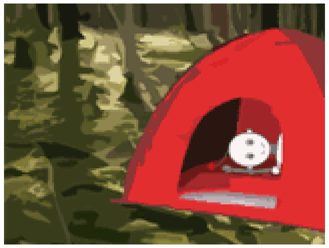
Camp in a Tent (10)

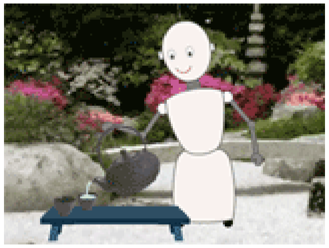
Drink Tea (5)

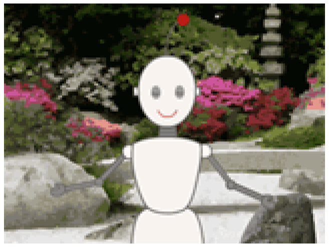

Meditate (20)

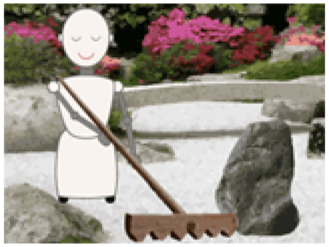

Rake the Sand (10)

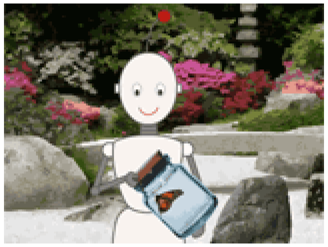

Release a Butterfly (25)

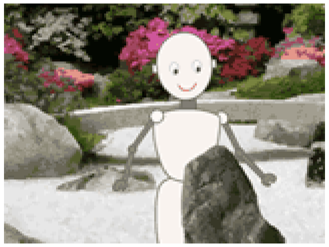

Arrange the Garden (15)

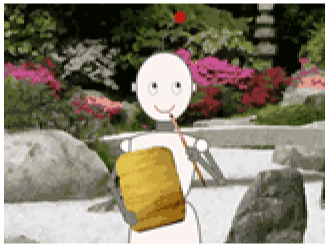

Write a Poem (10)

<u>**COLLECTIBLES organized by LOCATION**</u>

My Backyard

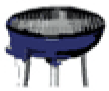
BBQ

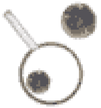
Bubble Wang

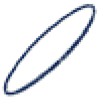
Hulahoop

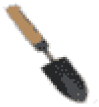
Spade

Moonstone Beach

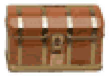
Treasure Chest

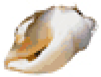
Seashell

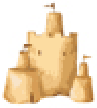
Sand Castle

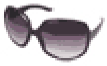
Sunglasses

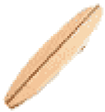
Surfboard

Crystal Cave

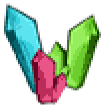
Crystals

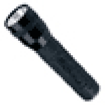
Flashlight

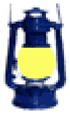
Lantern

Wellnesstown City Park

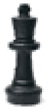
Chess Piece

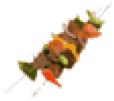
Skewers

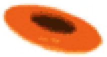
Frisbee

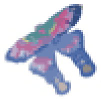
Kite

Blue Coyote Desert

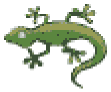
Lizard

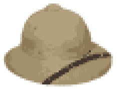
Hat

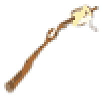
Marshmallow

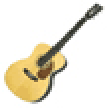
Guitar

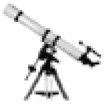
Telescope

Sunflower Valley

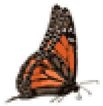
Butterfly

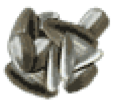
Seed

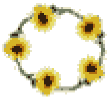
Flower Necklace

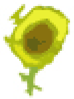
Sunflower

Red Cactus Mountain

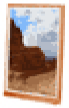
Painting

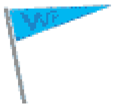
Flag

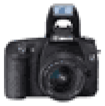
Camera

Sunny Hill Playground

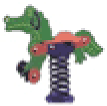
Spring Animal

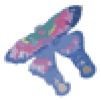
Kite

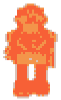
Toy

Shimmering Rock River	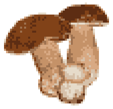 *Mushrooms*	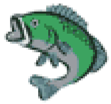 *Fish*	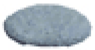 *Stone*	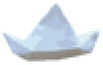 *Paper Boat*	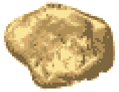 *Gold Nugget*
Golden Pines Woods	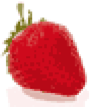 *Berry*	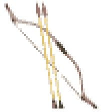 *Bow and Arrow*	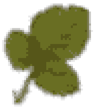 *Leaf*	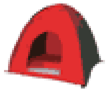 *Tent*	
Zen Garden	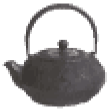 *Tea Kettle*	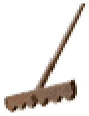 *Rake*	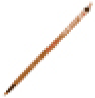 *Pen*	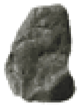 *Rock*	
