# Supplementary material for: Wellness Partners: Design and Evaluation of a Web-Based Physical Activity Diary with Social Gaming Features for Adults
Source: JMIR Res Protoc. 2013 Feb 1;2(1):e10. doi: 10.2196/resprot.2132 (PMC3628151; doi:10.2196/resprot.2132)
Supplement: Supplementary file 3 [file resprot_v2i1e10_app3.pdf]

WP Study Part A - Instructions

Welcome to Wellness Partners online! You have been randomly assigned to start with Part A of the study.

Overview

In this part of the study, you will be using an online social network site (similar to Facebook) to share your physical activity information with the family and friends whom you invite to join the program with you. We ask that you try login and spend a few minutes on the Wellness Partners site every day. The study team will provide a daily email with website usage tips and a survey link for providing feedback on its features. This version of the site includes features for you to **Post an Update** and **Send a Message**. It also displays your **total points** and allows you to interact with a virtual animated character. There is an optional feature to receive reminders from the Wellness Partners site via Twitter (email or text messaging) if you haven't posted any updates within a 24 hour period.

First Login:

1. Logon to http://wp.usc.edu/login with user *username* and password *password*
2. In the first screen, you will be asked to enter your **Twitter** username (optional), a **nickname** to use in this program, a new **password** and a name for your **virtual character**. You can change all these later from the **Preferences** section.

What can I do on the website?

- **Post an Update**: Each day, you can report completed physical activities (e.g., walking, running, doing chores) or setbacks (e.g., I was tired, I was busy). When reporting a particular physical activity, you can specify duration (15-90 minutes), intensity (light, medium, heavy) and mark it private (optional).
- **Earn Points**: You earn points for reporting completed physical activities and setbacks. You can earn more points for reporting a physical activity you completed *with your invited study partner(s)* and/or for posting updates on a regular basis.
- **Send Messages**: You can send direct messages to any of your study partner(s).
- **Review Daily Summary & Complete Activity History**: Every post you make will be displayed in the **Daily Summary** and the **Complete Activity History**. The Daily Summary resets on a daily basis. The Completed Activity History shows a detailed summary of posts from the beginning of the study. Your posts will be shared with your invited study partner(s) unless you mark them as private.
- **Review Partner Status**: Based on your selection under **Preferences** you can display to your study partner(s) when was the last time you logged on and how many points you have (Full Details), you can withhold your points (Hide Points) or you can hide all status information (Invisible).
- **Popular Activities**: Every new type of activity you and your study partner(s) log will be displayed in this area. Popular activities are displayed in a larger font than less popular activities. Popular activities can also be used as shortcuts for doing a quickpost.

What can I do with my points?
Your virtual character has a choice of free **Locations** they can visit with different **Activities** that can be redeemed for points. Once you pay for an activity using your points, you earn a **Memory** that you can replay unlimited times by clicking on it. Some Activities result in collectible items that get added to **Your Collection**. Collectibles can be gifted to your study partners by clicking on them.

Optional Twitter Feature
Twitter (http://www.twitter.com/) is a very popular social networking site. If you enable Twitter with Wellness Partners you will receive a daily email reminder via Twitter to post an update.

How do I enable Twitter with Wellness Partners?

- Make a new twitter account or use your existing account. You can enter your Twitter account information at first login or at any time by clicking on **Preferences**.
- In Twitter, search and follow "wp_bot". It will follow you back soon!

How do I enable Twitter Mobile Updates with Wellness Partners?

- Please review this document to setup your phone with Twitter if you haven't done this already: http://twitter.zendesk.com/forums/59008/entries/14014
- In Twitter, click on "following", find "wp_bot" and click on the **settings** icon to enable updates to be sent on your mobile phone via text messaging.

Getting to know Twitter
For help on Twitter, please read the following online document:
http://twitter.zendesk.com/forums/10711/entries/13920

Text Messaging Costs
Twitter is free, but text messaging on your cell phone may cost you a few cents per message depending on your cell phone plan. We will compensate you a flat fee of $15 if you complete the entire study to cover any text messaging costs regardless of whether you use the feature or not.

Wellness Partners Website Help/Feedback
If you are having trouble with the website or would like to give us positive or negative feedback about the software, please fill out the form below:
https://usc.qualtrics.com/SE?SID=SV_4TNTBBrishbdPjm&SVID=Prod

WP Study Part B – Instructions

Welcome to Wellness Partners online! You have been randomly assigned to start with Part B of the study.

Overview

In this part of the study, you will be using an online social network site (similar to Facebook) to share your physical activity information with the family and friends whom you invite to join the program with you. We ask that you try login and spend a few minutes on the Wellness Partners site every day. The study team will provide a daily email with website usage tips and a survey link for providing feedback on its features. This version of the site includes features for you to **Post an Update** and **Send a Message**. There is an optional feature to receive reminders from the Wellness Partners site via Twitter (email or text messaging) if you haven't posted any updates within a 24 hour period.

First Login:

1. Logon to http://wp.usc.edu/login with user *username* and password *password.*
2. Change your password by clicking on the **Preferences** link after your log on.

What can I do on the website?

- **Post an Update**: Each day, you can report completed physical activities (e.g., walking, running, doing chores) or setbacks (e.g., I was tired, I was busy). When reporting a particular physical activity, you can specify duration (15-90 minutes), intensity (light, medium, heavy) and mark it private (optional).
- **Send Messages**: You can send direct messages to any of your study partner(s).
- **Review Daily Summary & Complete Activity History**: Every post you make will be displayed in the **Daily Summary** and the **Complete Activity History**. The Daily Summary resets on a daily basis. The Completed Activity History shows a detailed summary of posts from the beginning of the study. Your posts will be shared with your invited study partner(s) unless you mark them as private.
- **Review Partner Status**: Based on your selection under **Preferences** you can display to your study partner(s) when was the last time you logged on and how many points you have (Full Details), you can withhold your points (Hide Points) or you can hide all status information (Invisible).
- **Popular Activities**: Every new type of activity you and your study partner(s) log will be displayed in this area. Popular activities are displayed in a larger font than less popular activities. Popular activities can also be used as shortcuts for doing a quickpost.

Optional Twitter Feature
Twitter (http://www.twitter.com/) is a very popular social networking site. If you enable Twitter with Wellness Partners you will receive a daily email reminder via Twitter to post an update.

How do I enable Twitter with Wellness Partners?

- Make a new twitter account or use your existing account. You can enter your Twitter account information at first login or at any time by clicking on **Preferences**.
- In Twitter, search and follow "wp_bot". It will follow you back soon!

How do I enable Twitter Mobile Updates with Wellness Partners?

- Please review this document to setup your phone with Twitter if you haven't done this already: http://twitter.zendesk.com/forums/59008/entries/14014
- In Twitter, click on "following", find "wp_bot" and click on the **settings** icon to enable updates to be sent on your mobile phone via text messaging.

Getting to know Twitter
For help on Twitter, please read the following online document:
http://twitter.zendesk.com/forums/10711/entries/13920

Text Messaging Costs
Twitter is free, but text messaging on your cell phone may cost you a few cents per message depending on your cell phone plan. We will compensate you a flat fee of $15 if you complete the entire study to cover any text messaging costs regardless of whether you use the feature or not.

Wellness Partners Website Help/Feedback
If you are having trouble with the website, or would like to give us positive or negative feedback about the software, please fill out the form below:
https://usc.qualtrics.com/SE?SID=SV_4TNTBBrishbdPjm&SVID=Prod
